# Precision of maximum likelihood estimation in adaptive designs

**DOI:** 10.1002/sim.6761

**Published:** 2015-10-12

**Authors:** Alexandra Christine Graf, Georg Gutjahr, Werner Brannath

**Affiliations:** ^1^ Medical University of ViennaCenter for Medical Statistics, Informatics and Intelligent SystemsSpitalgasse 231090ViennaAustria; ^2^ University of BremenCompetence Center for Clinical TrialsLinzer Strasse 428359BremenGermany

**Keywords:** maximum likelihood estimation, sample size reassessment, treatment selection, adaptive designs

## Abstract

There has been increasing interest in trials that allow for design adaptations like sample size reassessment or treatment selection at an interim analysis. Ignoring the adaptive and multiplicity issues in such designs leads to an inflation of the type 1 error rate, and treatment effect estimates based on the maximum likelihood principle become biased. Whereas the methodological issues concerning hypothesis testing are well understood, it is not clear how to deal with parameter estimation in designs were adaptation rules are not fixed in advanced so that, in practice, the maximum likelihood estimate (MLE) is used. It is therefore important to understand the behavior of the MLE in such designs. The investigation of Bias and mean squared error (MSE) is complicated by the fact that the adaptation rules need not be fully specified in advance and, hence, are usually unknown. To investigate Bias and MSE under such circumstances, we search for the sample size reassessment and selection rules that lead to the maximum Bias or maximum MSE. Generally, this leads to an overestimation of Bias and MSE, which can be reduced by imposing realistic constraints on the rules like, for example, a maximum sample size. We consider designs that start with *k* treatment groups and a common control and where selection of a single treatment and control is performed at the interim analysis with the possibility to reassess each of the sample sizes. We consider the case of unlimited sample size reassessments as well as several realistically restricted sample size reassessment rules. © 2015 The Authors. *Statistics in Medicine* Published by John Wiley & Sons Ltd.

## 1 Introduction

There has been increasing interest over the last years in adaptive two‐stage clinical trials where more than one treatment group are compared with one common control. These trials allow for design adaptations as, for example, sample size reassessment or treatment selection at an interim analysis. It is well known that ignoring the adaptive and multiplicity issues lead to a considerable inflation of the type 1 error rate and that effect estimates based on the maximum likelihood principle may be biased. For the comparison of a single treatment with a control and balanced sample sizes between groups, Proschan and Hunsberger 1 showed that the maximum type 1 error rate can be inflated from 0.05 to 0.11. Graf and Bauer 2 extended this arguments to allow for individual sample size reassessment rules in the treatment and control group respectively, which increases the maximum type 1 error to 0.19. However, when selecting one out of *k* treatments and control for a second stage, Graf *et al.*, 3 showed that if using the Dunnett test to adjust for multiplicity 4, the maximum type 1 error rate may not exceed the pre‐specified *α*‐level for specific restrictions on the second stage sample size reassessment rule because of the over‐correction for the treatments not tested at the end of the study. A large number of hypothesis testing methods have been developed that allow for flexible sample size adaptations (not pre‐fixed in advance) without compromising the overall type 1 error rate based on the combination test approach 5, 6, 7 or the conditional error principle 8, 9 and have been extended to multi‐armed clinical trials allowing for treatment selection 6, 7, 10, 11, 12.

Whereas the methodological issues concerning hypothesis testing are well understood, up to now, it is not clear how to deal with parameter estimation after flexible interim adaptations. Several methods have been proposed to reduce or remove the Bias 12, 13, 14, 15, 16, 17, 18, 19. The Bias depends on many different features as the selection procedure, the sample size reassessment rule, or the unknown parameters. The proposed methods therefore do only apply to specific adaptation rules and, hence, are not generally applicable. In particular, in designs were adaptation rules are not fixed in advance, estimation is still an unsolved issue, so that in practice the maximum likelihood estimate (MLE) is still used.

Bauer *et al*. 20 investigated the impact of treatment selection on the mean Bias and the mean squared error (MSE) when selecting those *j* (out of *k*) treatments with the largest observed effects while fixing the total per‐group sample size. They further considered designs where the sample size is reshuffled equally to the selected treatment arms and control with the conclusion that due to regression of the mean, Bias decrease as compared with the scenario without reshuffling. To our knowledge, no investigations of other types of sample size and selection rules where undertaken yet. Hence, the behavior of MLE is not yet fully understood for adaptive designs.

Adaptive designs have the practically important feature that the selection and sample size reassessment rule need not be fully pre‐specified. This complicates the investigation of Bias and MSE, which depend on the actually unknown sample size and selection rule. Simulations or numerically investigations under typical adaption rules are important, however, can only give partial answers. We therefore investigate the behavior of the MLE from another point of view; we search for the selection and sample size reassessment rule leading to the maximum mean Bias or maximum MSE when using the MLE at the end of the adaptive trial to estimate the treatment effect. Brannath *et al.*, 16 calculated the maximum mean Bias for the case of a one‐sample *z*‐test and concluded that the maximum mean Bias in a flexible two stage design is in general and not larger than that of a conventional group sequential design. We will consider scenarios where more than one treatment groups are compared with a common control, and one treatment and the control are selected for the second stage. Moreover, we also allow for flexible choices of the second stage allocation ratio, permitting, for example, a larger increase in sample size for the selected treatment than for the control group.

The case of unlimited sample sizes provides upper bounds for Bias and MSE. We therefore consider also scenarios with restrictions on the sample size to obtain less conservative estimates for real adaptive trials. We will, for instance, investigate bounded second stage sample sizes as well as the restriction on the control group to have a smaller sample size than that of the treatment group. We will also consider designs with a fixed overall sample size for the control group and designs with a fixed total sample size permitting only a reshuffling between the selected treatment and the control, with and without the restriction of a smaller control group.

We will see in this paper that the maximum mean Bias and maximum MSE of the MLE are independent from the true means in the treatment and control groups, without and with restrictions on the second stage sample sizes. As a consequence, they are the same under the null and all alternative hypothesis. This is a very attractive property of the maximum mean Bias and maximum MSE of the MLE that simplifies its investigation and discussion considerably.

The rest of the paper is organized as follows. In Section 2, we describe the type of interim adaptations investigated to calculate the maximum mean Bias and maximum MSE. In Section 3, we investigate the maximum mean Bias and maximum MSE for the case when only *k* = 1 treatment is compared with one control. In Section 4 we generalize the arguments to the scenario of selecting one out of *k* > 1 treatments and control for the second stage. A strategy that is intensively discussed in the literature 11, 12, 13, 18, 20. We will end with a discussion of the results in Section 5.

## 2 Designs with treatment selection

Assume a clinical trial with parallel groups and a two‐stage design that starts at the first stage with *k* treatments and a control and continues in the second stage with one selected treatment and the control. We assume normally distributed outcomes, *X*_(*i*,*j*,*l*)_∼*N*(*μ*_*i*_,*σ* ^2^), *i* = 0,…,*k*, where *i* represents the treatment group, with *i* = 0 for the control and *i* = 1,…,*k* for the experimental treatments, and *j*∈{1,2} is the index for the stage. The index *l* stands for the individual, where *l* = 1,…,*n*_(*i*,1)_ in the first stage and *l* = 1,…,*n*_(*i*,2)_ in the second stage for each treatment group *i*. The common variance *σ* ^2^ is assumed to be known.

An interim analysis is performed after recruitment of *n*_(*i*,1)_ patients in the *i*th experimental treatment and *n*_(0,1)_ in the control group. For simplicity, we assume balanced sample sizes in the first stage, that is, *n*_(*i*,1)_=*n*_(0,1)_=*n* for all *i* = 1,…,*k*, which is a common scenario. However, the second stage sample sizes can be unbalanced. Based on the data of the first stage, *X*_(*i*,1,*l*)_, *i* = 0,…,*k* and *l* = 1,…,*n*, we select one out of the *k* treatments, say treatment *s*∈{1,…,*k*}, and the control for the second stage. We may also reassess the second sample sizes based on the first stage data. In the second stage, *n*_(*s*,2)_=*r*_*s*_ *n*, patients are recruited in the selected treatment and *n*_(0,2)_=*r*_0_ *n* in the control group, where second‐to‐first‐stage ratios 0≤*r*_*i*_≤*∞* for *i* = 0,*s*, can depend on the first stage data. Note that the selected treatment (or control) can also be stopped at interim by setting *r*_*s*_=0 (or *r*_0_=0). In contrast to the majority of the literature on point estimation in designs with treatment selection, we do not assume a specific selection or sample size reassessment rule and thereby consider the full flexibility permitted with adaptive designs 6, 7, 10, 11.

Treatment selection here means to decide on the treatment ‘of interest’ for which the effect estimate will further be investigated. For treatments, not selected in the interim analysis, we assume that the treatment effect is not of interest at the end of the trial. In the final analysis, the overall effect of the selected treatment to control is calculated using the maximum likelihood estimators, calculated over both stages:

$${\bar{x}}_{i}=\frac{{\bar{x}}_{\left(i,1\right)}+r_{i}{\bar{x}}_{\left(i,2\right)}}{1+r_{i}},$$

where *i* = 0,*s*, ${\bar{x}}_{\left(i,1\right)}=\frac{1}{n}\overset{n}{\underset{l=1}{\sum }}x_{\left(i,1,l\right)}$ is the sample mean of the first stage, and ${\bar{x}}_{\left(i,2\right)}$ is the sample mean of the second stage for group *i* = 0,*s*. If sample size adjustments are performed based on the first stage data, the overall sample mean ${\bar{x}}_{i}$ may be biased (see e.g. Brannath *et al*. 16).

Our intention is to derive the worst case, meaning that we are searching for the sample size reassessment and selection rule maximizing the mean Bias (denoted in sequel as "Bias" for short) or the MSE for the selected treatment compared to the control. We prefer to consider the ‘root mean squared error’, $\text{RMSE}=\sqrt{\text{MSE}}$, because it is on the same scale as the mean and the Bias. In the context of designs with treatments selection, the Bias and MSE are defined as follows:

$$\text{Bias}=\text{E}\left[\left({\bar{X}}_{s}-{\bar{X}}_{0})-(\mu_{s}-\mu_{0}\right)\right]\;\text{and}\;\text{MSE}=\text{E}\left[{\left(({\bar{X}}_{s}-{\bar{X}}_{0})-(\mu_{s}-\mu_{0})\right)}^{2}\right].$$

These quantities have also been denoted by ‘selection Bias’ and ‘selection MSE’ (cf. Bauer *et al*. 20).

The general idea of this paper is to determine the maximum Bias or maximum MSE by maximizing at each interim sample point the conditional Bias or conditional MSE given the interim data. By searching for the treatment selection and sample size adaptation rules that maximizes the conditional Bias (or MSE), we obtain the treatment selection and sample size rules that maximizes the overall Bias (or MSE). This idea has been used in Brannath *et al.*, 16 to obtain the maximum Bias in the one‐sample case and, thereby, also in the balanced two‐sample case. A similar idea has earlier (and later) been used to determine the maximum type 1 error rate of the naive *z*‐test or Dunnett‐test 1, 2, 3.

## 3 Two‐arm trials with sample size reassessment

For illustrative purposes, we start with the scenario where only one treatment group (*k* = *s* = 1) is compared with a control. The results will be generalized to *k* > 1 in Section 4. We start with a discussion of the maximum Bias and then proceed with a similar investigation of the maximum RMSE.

### 3.1 Maximum Bias

Brannath *et al.*, 16 calculated the maximum Bias of the one‐sample mean in a two‐stage design with data‐driven sample size reassessments. Their result easily generalizes to the treatment effect estimate in a two‐arm parallel group design with balanced first and second stage sample sizes, because the treatment effect estimate in a balanced two‐arm trial is formally equal to the one‐sample mean of observations with variance 2*σ* ^2^. According to the result in 16, the maximum Bias in an adaptive two‐stage trial with two‐arms and the restriction *n*_(0,2)_=*n*_(1,2)_ becomes

(1) $${\text{B}}^{*}(n,\sigma ,r_{\min},r_{\max})=\frac{\sqrt{2}\sigma }{\sqrt{n}}\phi (0)\left(\frac{1}{1+r_{\min}}-\frac{1}{1+r_{\max}}\right)\approx \frac{0.6\;\sigma }{\sqrt{n}}\left(\frac{1}{1+r_{\min}}-\frac{1}{1+r_{\max}}\right)$$

where *ϕ* denotes the standard normal density and *r*_min_ and *r*_max_ are pre‐specified lower and upper bounds for the data driven second‐to‐first‐stage ratio *r* = *n*_(0,2)_/*n* = *n*_(1,2)_/*n*. Note that the maximum Bias is independent from the true means *μ*_0_ and *μ*_1_. We can set *r*_min_=0 and *r*_max_=*∞* if no such bounds exist. In this case, the maximum Bias becomes ${\text{B}}^{*}(n,\sigma ,0,\infty )=\phi (0)\sqrt{2}\;\sigma /\sqrt{n}\approx 0.6\;\sigma /\sqrt{n}$.

#### 3.1.1 Flexible second‐to‐first‐stage ratios

The restriction to *n*_(0,2)_=*n*_(1,2)_ may be too strong for applications because it does not permit an unequal increase or decrease of the sample sizes in the two arms. For instance, if the control is a placebo, ethical reasons may advise us to increase the sample size only in the treatment group or even decrease it in the control arm. A reduction in the placebo allocation ratio will usually also increase the willingness to participate in the trial. We, therefore, also consider the Bias under unequal sample size adaptations, which can be determined by maximizing the conditional Bias with regard to *n*_(0,2)_ and *n*_(1,2)_ without the constraint *n*_(0,2)_=*n*_(1,2)_. We will see in subparagraph 3.1.5 (where we describe our calculations) that the maximum Bias remains independent from *μ*_1_ and *μ*_0_ for flexible second stage sample sizes *n*_(0,2)_ and *n*_(1,2)_. Note that *r*_0_=*n*_(0,2)_/*n* and *r*_1_=*n*_(1,2)_/*n* are the individual second‐to‐first‐stage ratios for the control and treatment group with *r*_min_≤ min(*r*_0_,*r*_1_)≤ max(*r*_0_,*r*_1_)≤*r*_max_.

**Figure 1:**
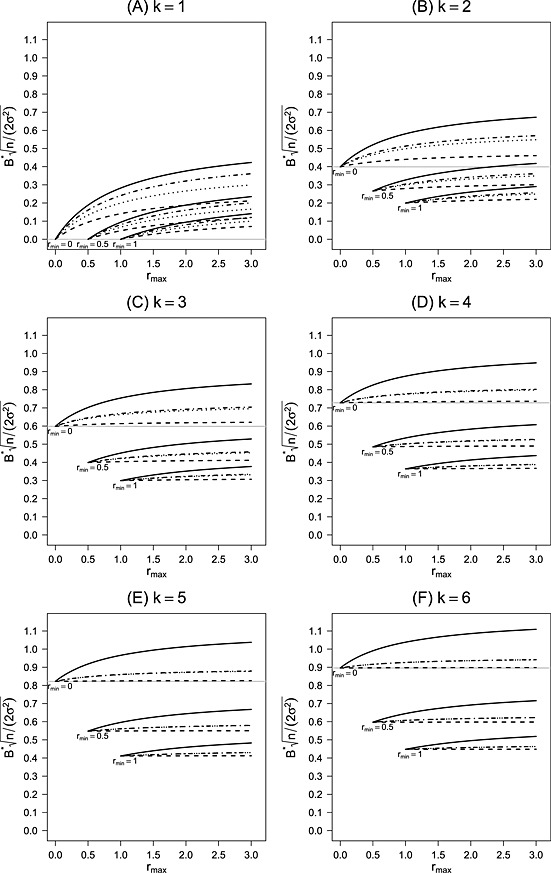
Standardized maximum Bias as a function of *r*_max_ for *r*_min_=0, 0.5, and 1. Values are given for a number of *k* = 1 to 6 treatments in panels (A) to (F). Within one panel the standardized maximum Bias is shown for different restrictions on the sample size reassessment rule: flexible second‐to‐first‐stage‐ratios (solid lines), equal second‐to‐first‐stage ratios (dotted lines), restricting $r_{1}\geq r_{0}$ (dot‐dashed lines), and fixing the control (dashed lines). The gray horizontal line shows the standardized Bias for a fixed‐size‐sample test with post‐trial selection.

Figure 1 (A) for *k* = 1 shows the maximum Bias, B^∗^, standardized by the standard error $\sqrt{2\sigma^{2}/n}$ of the first‐stage mean. The shown results do therefore also not depend on the first stage sample size *n* or the common known variance *σ*. The solid lines in Figure 1 (A) show ${\text{B}}^{*}/\sqrt{2\sigma^{2}/n}$ for *r*_min_=0,0.5, and 1 and *r*_max_ varying from 0 to 3. As expected, the maximum Bias is increasing with decreasing *r*_min_ and increasing *r*_max_, showing that more flexibility leads to a larger maximum Bias. For example, for *r*_min_=1, meaning that the second‐stage sample size has to be as least as large as the first‐stage sample size and *r*_max_=2 allowing a doubling of the second‐stage sample size as compared with the first stage, the maximum Bias is 0.09 times the first‐stage standard error, increasing to 0.19 and 0.38 for *r*_min_=0.5 and 0, respectively. This shows that the option for sample size reductions (including early stopping) can largely increases B^∗^.

The maximum Bias appears to be large for some of the scenarios in Figure 1 (A). However, recall that we have plotted B^∗^ in units of $\sqrt{2\sigma^{2}/n}$ and that $\sqrt{2\sigma^{2}/n}$ decreases with increasing per group first‐stage sample size *n*. Assume, for instance, that *n* is half the sample size required for a *z*‐test with power 90% at *δ* = *μ*_*i*_−*μ*_0_ in a classical two‐armed parallel group design at one‐sided level *α* = 0.025. Then $\sqrt{2\sigma^{2}/n}=\delta {\left\{\Phi^{-1}(0.975)+\Phi^{-1}(0.9)\right\}}^{-1}=0.31\;\delta$, and if (*r*_min_,*r*_max_) = (1,2), then ${\text{B}}^{*}=0.09\;\sqrt{2\sigma^{2}/n}$ is only 3*%* of the effect size *δ* assumed in the sample size calculation. Allowing for more flexibility, as, for example, (*r*_min_,*r*_max_) = (0,2), the bias is substantially increasing to 12*%* of the effect size.

#### 3.1.2 Restriction to $r_{1}\geq r_{0}$

A reasonable constraint to reduce the maximum Bias is to require that the experimental treatment group is never smaller than the control group. In this case, *r*_0_ can vary between (*r*_min_,*r*_max_), while *r*_1_ is restricted to (*r*_0_,*r*_max_). The dot‐dashed lines in Figure 1 (A) show the standardized maximum Bias for this type of restriction. As expected, the maximum Bias is always smaller than the maximum Bias with flexible ratios but larger than the one with balanced second‐stage sample sizes. Its line is right in the middle of the two other lines. The dotted lines in Figure 1 (A) shows the standardized maximum Bias if we restrict the second‐stage sample sizes to be balanced. If *r*_max_=2, the standardized maximum Bias under the given constraint becomes 0.08, 0.16, and 0.32 for *r*_min_=1, 0.5, and 0, respectively. We can see that for $r_{\min}\geq 0.5$ the difference in Bias between the constraints *r*_1_=*r*_0_ and $r_{1}\geq r_{0}$ is small.

#### 3.1.3 Fixing *r*_0_

A stronger restriction is to fix the total sample size of the control group (that is, fixing *r*_0_) while in the experimental treatment group sample sizes are reassessed within the window (*r*_0_,*r*_max_). The MLE of the control group is then unbiased. The maximum Bias, therefore, does not depend on the interim outcome of the control group. However, it depends on the fixed *r*_0_. The dashed lines in Figure 1 (A) give the standardized maximum Bias for *r*_0_=0,0.5, or 1 while *r*_max_ varies from *r*_0_ to 3. For example, if *r*_0_=1 and *r*_max_=2, then the standardized maximum Bias is 0.05, that is, a little more than half of the Bias with flexible sample size reallocations. We are aware that fixing *r*_0_=0 may be an unrealistic scenario always resulting in a second stage without control. However, for complete presentation of the results, the Figure also shows the line for *r*_0_=0.

#### 3.1.4 The effect of *r*_min_

Figure 1 (A) indicates that the minimum *r*_min_ for the second‐stage sample sizes has quite some impact on the maximum Bias. To further elaborate the impact of *r*_min_, we have calculated the maximum Bias for *r*_max_=*∞* and *r*_min_=0,0.5,1. Table 1 shows the results for the standardized maximum Bias. The row ‘flexible’ gives the maximum Bias with flexible second‐stage allocation ratios; in the row ‘ $r_{1}\geq r_{0}$’, the sample size of the experimental treatment group is constraint to be at least as large as in the control group. ‘*r*_0_=*r*_1_’ means that the second‐stage sample sizes are restricted to be balanced, and ‘fix *r*_0_’ that the sample size of the control group is fixed. Here, we are interested in the case *k* = 1 (one experimental treatment only). We observe that the maximum Bias is halved by letting *r*_min_=1 (i.e. forcing the second‐stage sample sizes to be at least as large as the first‐stage ones) compared with *r*_min_=0. With *r*_min_=0.5, the maximum Bias is reduced by about 33%. These factors for the maximum Bias seem to be completely independent from the additional restrictions on *r*_1_ and *r*_0_ which is a remarkable finding. A possible explanation for this finding is that the maximum bias is dominated by the minimum sample size *r*_min_.

**Table 1:** The standardized maximum Bias, ${\text{B}}_{k}^{*}\sqrt{n/(2\sigma^{2})}$, as well as the standardized maximum root mean squared error, $\sqrt{{\text{MSE}}_{k}^{*}(n/(2\sigma^{2}))}$, for different restrictions for the sample size reassessment rules: flexible *r*_*s*_ and *r*_0_, balanced second‐stage sample size (*r*_*s*_=*r*_0_), a larger second stage sample size in the treatment group ( $r_{s}\geq r_{0}$) and fixing the sample size in the control (fix *r*_0_). Values are given for *r*_min_=0, 0.5 and 1 setting *r*_max_=*∞*. For comparison, the fixed design with *r*_min_=*r*_max_ is given showing the maximum selection Bias and mean squared error, respectively.

*k*	type	*r*_min_					
		${\text{B}}_{k}^{*}\sqrt{n/(2\sigma^{2})}$			$\sqrt{{\text{MSE}}_{k}^{*}n/(2\sigma^{2})}$		
		0	0.5	1	0	0.5	1
1	*r*_min_=*r*_max_	0.000	0.000	0.000	1.000	0.817	0.707
	flexible	0.564	0.376	0.282	1.129	0.859	0.723
	$r_{1}\geq r_{0}$	0.482	0.321	0.241	1.092	0.843	0.717
	*r*_1_=*r*_0_	0.399	0.266	0.199	1.039	0.820	0.707
	fix *r*_0_	0.282	0.188	0.141	1.080	0.842	0.717
2	*r*_min_=*r*_max_	0.399	0.266	0.199	1.246	0.955	0.799
	flexible	0.764	0.509	0.382	1.320	0.980	0.809
	$r_{s}\geq r_{0}$	0.628	0.419	0.314	1.276	0.963	0.801
	*r*_*s*_=*r*_0_	0.598	0.399	0.299	1.258	0.956	0.799
	fix *r*_0_	0.482	0.321	0.241	1.271	0.962	0.801
3	*r*_min_=*r*_max_	0.598	0.399	0.299	1.389	1.040	0.856
	flexible	0.910	0.607	0.455	1.446	1.059	0.864
	$r_{s}\geq r_{0}$	0.739	0.493	0.370	1.402	1.042	0.857
	*r*_*s*_=*r*_0_	0.728	0.485	0.364	1.395	1.040	0.856
	fix *r*_0_	0.628	0.419	0.314	1.399	1.042	0.857
4	*r*_min_=*r*_max_	0.728	0.485	0.364	1.489	1.099	0.897
	flexible	1.022	0.681	0.511	1.537	1.117	0.904
	$r_{s}\geq r_{0}$	0.827	0.551	0.414	1.495	1.100	0.897
	*r*_*s*_=*r*_0_	0.882	0.548	0.411	1.492	1.099	0.897
	fix *r*_0_	0.739	0.493	0.370	1.493	1.100	0.897
5	*r*_min_=*r*_max_	0.822	0.548	0.411	1.565	1.145	0.929
	flexible	1.109	0.739	0.555	1.608	1.161	0.935
	$r_{s}\geq r_{0}$	0.898	0.599	0.449	1.567	1.146	0.929
	*r*_*s*_=*r*_0_	0.896	0.597	0.488	1.566	1.145	0.929
	fix *r*_0_	0.827	0.551	0.414	1.567	1.145	0.926
6	*r*_min_=*r*_max_	0.895	0.597	0.448	1.625	1.181	0.954
	flexible	1.180	0.787	0.590	1.666	1.197	0.960
	$r_{s}\geq r_{0}$	0.957	0.638	0.479	1.627	1.182	0.954
	*r*_*s*_=*r*_0_	0.956	0.637	0.478	1.627	1.181	0.954
	fix *r*_0_	0.898	0.599	0.449	1.626	1.182	0.954

#### 3.1.5 Determination of maximum Bias

As mentioned in the introduction, the sample size reassessment rule, which maximizes the Bias, is obtained by maximizing the conditional Bias, that is, the deviation of the conditional mean of the treatment effect estimate (given the interim data) from the true parameter value.

For the calculation of the conditional Bias, we standardize the individual stage‐wise means $Z_{\left(i,j\right)}=({\bar{X}}_{\left(i,j\right)}-\mu_{i})\sqrt{n_{\left(i,j\right)}/\sigma^{2}}$, *i* = 0,1, *j* = 1,2. Recall that *n*_(1,1)_=*n*_(0,1)_=*n* and our definition of *r*_*i*_=*n*_(*i*,2)_/*n*, *i* = 0,1, with *r*_min_≤ min(*r*_0_,*r*_1_)≤ max(*r*_0_,*r*_1_)≤*r*_max_. To simplify the notation, we will omit the index *j* for the first stage data and summaries, for example, denoting the first‐stage standardized means by *z*_*i*_, *i* = 0,1. Similar calculations as in 16 give the conditional Bias:

(2) $$\begin{array}{cc} \text{CB}(z_{0},z_{1},r_{0},r_{1},n,\sigma ) & =\text{E}\left[({\bar{X}}_{1}-{\bar{X}}_{0})-(\mu_{1}-\mu_{0})|Z_{\left(i,1\right)}=z_{i},i=0,1\right]\\ =\frac{\sigma }{\sqrt{n}}\left[\frac{z_{1}}{1+r_{1}}-\frac{z_{0}}{1+r_{0}}\right]. \end{array}$$

To evaluate the worst case, the second‐to‐first‐stage ratios *r*_1_ and *r*_0_ are searched to maximize (2):

(3) $$\tilde{\text{CB}}(z_{0},z_{1},n,\sigma ,r_{\min},r_{\max})=\underset{r_{\min}\leq r_{0},r_{1}\leq r_{\max}}{\max}\text{CB}(z_{0},z_{1},r_{0},r_{1},n,\sigma )$$

Note again that we assume in the following the same lower and upper bounds *r*_min_,*r*_max_ for *r*_0_ and *r*_1_ and that (3) corresponds to the fully flexible case without additional restrictions on *r*_0_ and *r*_1_ (like e.g. $r_{1}\geq r_{0}$). The generalization to different boundaries and additional restrictions on (*r*_1_,*r*_0_) are formally straightforward. Clearly, $\tilde{\text{CB}}$ depends on the restrictions made for the ratios *r*_*i*_.

To assess the worst case reassessment rule for a given interim result, the true means of treatment and control group have to be known. However, our intention is to evaluate an upper bound for the overall Bias. The maximum Bias B^∗^ is evaluated by integrating the maximum conditional Bias over all interim outcomes:

(4) $${\text{B}}^{*}(n,\sigma ,r_{\min},r_{\max})=\overset{\infty }{\underset{-\infty }{\int }}\overset{\infty }{\underset{-\infty }{\int }}\tilde{\text{CB}}(z_{0},z_{1},n,\sigma ,r_{\min},r_{\max})\phi (z_{1})\phi (z_{0})dz_{1}dz_{0}\;.$$

Obviously, the maximum Bias B^∗^(*n*,*σ*,*r*_min_,*r*_max_) does not depend on the unknown *μ*_0_ and *μ*_1_.

Fortunately, (2) is the sum of two terms depending only on *r*_1_ or *r*_0_. Hence, in the fully flexible case, we can maximize each term separately in *r*_1_ or *r*_0_, respectively. Denoting the worst case sample size fractions by ${\tilde{r}}_{i}$, *i*∈{0,1}, we obtain ${\tilde{r}}_{1}=r_{\min}$ for *z*_1_>0 and ${\tilde{r}}_{1}=r_{\max}$ for *z*_1_<0. Similarly, ${\tilde{r}}_{0}=r_{\max}$ for *z*_0_>0 and ${\tilde{r}}_{0}=r_{\min}$ for *z*_0_<0. Figure (A) in the Appendix shows the four subsets of the interim outcome space corresponding the four values of the tuple $\left({\tilde{r}}_{0},{\tilde{r}}_{1}\right)$. The maximum Bias, B^∗^, is obtained by integrating the maximum conditional Bias in each subset and summing up the four integrals. This leads to

(5) $${\text{B}}^{*}(n,\sigma ,r_{\min},r_{\max})=\frac{2\sigma }{\sqrt{n}}\phi (0)\left[\frac{1}{1+r_{\min}}-\frac{1}{1+r_{\max}}\right]\approx \frac{0.8\sigma }{\sqrt{n}}\left[\frac{1}{1+r_{\min}}-\frac{1}{1+r_{\max}}\right]\;.$$

Without any restrictions on the second‐stage sample size reassessment rule, that is, setting (*r*_min_,*r*_max_) = (0,*∞*), the maximum Bias simplifies to ${\text{B}}^{*}(n,\sigma ,0,\infty )=2\phi (0)\sigma /\sqrt{n}\approx 0.8\sigma /\sqrt{n}$. Comparison of (1) and (5) reveals, that when dropping the constraint of equal second‐stage sample sizes, the maximum Bias is increased by the factor $\sqrt{2}$, that is, by about 41*%*.

We finally note how to account for constraints like $r_{1}\geq r_{0}$. To account for $r_{1}\geq r_{0}$, we need to rule out that *r*_1_=*r*_min_ and *r*_0_=*r*_max_. For *z*_0_>0 and *z*_1_>0, we therefore maximize CB(*z*_0_,*z*_1_,*r*_0_,*r*_1_,*n*,*σ*) under the assumption *r*_1_=*r*_0_. In this case, the maximum depends on *z*_1_−*z*_0_: it is attained for *r*_1_=*r*_0_=*r*_min_ if *z*_1_−*z*_0_>0 and otherwise for *r*_1_=*r*_0_=*r*_max_. The maximization of CB(*z*_0_,*z*_1_,*r*_0_,*r*_1_,*n*,*σ*) under the constraint of a fixed *r*_0_ follows similar lines as in the fully flexible case (leading to a rule that depends on *z*_1_ only).

#### 3.1.6 Reshuffling

Assume now that a sample size of *n*_*g*_ patients per group is pre‐planned over both stages, resulting in a total of 2*n*_*g*_ patients in the trial. This overall patient number is kept fix. The interim analysis is performed after recruitment of *t* *n*_*g*_ patients per group where *t*∈(0,1). The ratio *t* can be interpreted as the timing of the interim analysis. To keep the overall sample size, the second stage needs to consist of 2(1 − *t*)*n*_*g*_ patients in total. This number is allocated to the experimental treatment and control group in a data dependent manner. This means that in the interim analysis, a second‐stage sample size allocation rate *v*, 0≤*v*≤1, is chosen based on the interim results, such that in the second stage a number of *v*2(1 − *t*)*n*_*g*_ patients is allocated to the control and (1 − *v*)2(1 − *t*)*n*_*g*_ to the experimental treatment group. The conditional Bias (2) can be rewritten as follows:

(6) $$\text{CB}(z_{0},z_{1},v,n_{g},t,\sigma )=\frac{\sigma }{\sqrt{tn_{g}}}\left[\frac{z_{1}}{1+(1-v)w_{t}}-\frac{z_{0}}{1+vw_{t}}\right]$$

where we use the notation $w_{t}=2(\frac{1}{t}-1)$ for mathematical convenience. The allocation ratio 0≤*v*≤1 is now searched to maximize the conditional Bias (6). By setting the first derivative of the conditional Bias to zero, we obtain a quadratic equation with the two roots

$$v^{\left(1),(2\right)}=\frac{-z_{1}+z_{0}(w_{t}+1)\pm \sqrt{-z_{0}z_{1}}(w_{t}+2)}{(z_{0}+z_{1})w_{t}}$$

that are candidates for the $\tilde{v}$ maximizing the conditional Bias. The candidates *v* ^(1)^ and *v* ^(2)^ do only exist if *z*_0_ and *z*_1_ have different signs and *z*_0_≠−*z*_1_. If *z*_0_=−*z*_1_>0, one can see from (6) that the conditional Bias is maximized for *v* ^(3)^=1/2. Furthermore, *v* ^(1)^ and *v* ^(2)^ are ineligible if larger than 1 or smaller than 0. Whether *v* ^(1)^ or *v* ^(2)^ is actually the maximizer depends on *z*_0_ and *z*_1_. To assess the global maximum, the candidates *v* ^(4)^=0 and *v* ^(5)^=1 also have to be investigated. Note that for *z*_0_=−*z*_1_<0, candidates *v* ^(4)^ and *v* ^(5)^ coincide and show the maximizer of the conditional Bias. The worst case conditional Bias is the maximum of the five candidates.

(7) $$\tilde{\text{CB}}(z_{0},z_{1},n_{g},t,\sigma )=\underset{i=1,...,5:0\leq v^{\left(i\right)}\leq 1}{\max}\left[\text{CB}(z_{0},z_{1},v^{\left(i\right)},n_{g},t,\sigma )\right].$$

Figure (B) in the Appendix shows the subspaces of the interim outcome in terms of the standardized means in the treatment and control groups corresponding to the different maximizer *v* ^(*i*)^ for *t* = 0.5. The white area gives the subspace where either *v* ^(1)^ or *v* ^(2)^ are the global maximum. The dashed line gives the subspace, where *v* ^(3)^ is the global maximum. It can be seen that *v* ^(1)^ is no global optimum for *t* = 0.5. Numerical integration can be used to compute the overall Bias:

(8) $${\text{B}}^{*}(n_{g},t,\sigma )=\overset{\infty }{\underset{-\infty }{\int }}\overset{\infty }{\underset{-\infty }{\int }}\tilde{\text{CB}}(z_{0},z_{1},n_{g},t,\sigma )\phi (z_{1})\phi (z_{0})dz_{1}dz_{0}.$$

For numerical integration, we used the R‐package R2Cuba 21. In the following, we also show results under the restriction 0≤*v*≤0.5, which guarantees that the second‐stage sample size of the experimental treatment group is never smaller than that of the control group. The solid black line, marked with 1 in Figure 3 (A), shows the standardized maximum Bias as a function of the timing of the interim analysis *t* for *k* = 1 and 0≤*v*≤1. The maximum Bias is now standardized by the standard error of a fixed‐size‐sample test with per group sample size *n*_*g*_, that is, $\sqrt{2\sigma^{2}/n_{g}}$. We are not standardizing with the standard error of the interim estimate because it depends on *t*. The dashed line (marked with 1) gives the standardized maximum Bias under the restriction 0≤*v*≤0.5. One can see that (for *k* = 1) the standardized maximum Bias is decreasing with increasing *t*, that is, the later interim analysis, the smaller the maximum Bias. This is due to the larger first and smaller second‐stage sample sizes. For *t* = 0.5, that is, planning the interim analysis half way, the standardized maximum Bias is 0.40 if 0≤*v*≤1 and decreases to 0.21 if 0≤*v*≤0.5.

### 3.2 Maximum mean squared error

To maximize the MSE, we proceed similar to calculating the maximum Bias. For each interim outcome, the sample size reassessment rule is searched that maximize the conditional MSE (worst case). The conditional MSE, given the interim outcome, can be calculated as follows (see Appendix A.1):

(9) $$\begin{array}{cc} \text{CMSE}(z_{0},z_{1},r_{0},r_{1},n,\sigma ) & =\text{E}\left[{\left(\left({\bar{X}}_{1}-{\bar{X}}_{0})-(\mu_{1}-\mu_{0}\right)\right)}^{2}|Z_{\left(i,1\right)}=z_{i},i=0,1\right]\\ =\frac{\sigma^{2}}{n}\left[{\left(\frac{z_{1}}{1+r_{1}}-\frac{z_{0}}{1+r_{0}}\right)}^{2}+\frac{r_{1}}{{\left(1+r_{1}\right)}^{2}}+\frac{r_{0}}{{\left(1+r_{0}\right)}^{2}}\right] \end{array}$$

For each *z*_0_ and *z*_1_, *r*_0_ and *r*_1_ are searched to maximize the CMSE:

(10) $$\tilde{\text{CMSE}}\left(z_{0},z_{1},n,\sigma ,r_{\min},r_{\max}\right)=\underset{r_{\min}\leq r_{0},r_{1}\leq r_{\max}}{\max}\text{CMSE}\left(z_{0},z_{1},n,\sigma ,r_{0},r_{1}\right)$$

where *r*_min_ and *r*_max_ again denote the lower and upper bounds for the second‐to‐first‐stage ratios *r*_*i*_, *i* = 0,1. Additional constraints on (*r*_0_,*r*_1_) like $r_{1}\geq r_{0}$ need to be accounted in the maximum (10). Integrating over all interim outcomes gives the maximum MSE, denoted by MSE^∗^ in the sequel,

(11) $${\text{MSE}}^{*}(n,\sigma ,r_{\min},r_{\max})=\overset{\infty }{\underset{-\infty }{\int }}\overset{\infty }{\underset{-\infty }{\int }}\tilde{\text{CMSE}}(z_{0},z_{1},n,\sigma ,r_{\min},r_{\max})\phi (z_{0})\phi (z_{1})dz_{0}dz_{1}\;.$$

Note that MSE^∗^ is also independent from the group means *μ*_*i*_, *i* = 0,1.

#### 3.2.1 Flexible second‐to‐first‐stage ratios

We start investigating the case of completely flexible *r*_1_ and *r*_0_ within the boundaries (*r*_min_,*r*_max_). Note again that we assume equal bounds for the treatment and the control group; however, the sample size reassessment rule for the treatment and control group can be different. To maximize the CMSE in (9), for given *z*_0_ and *z*_1_ at interim, a total of nine candidates have to be investigated, and the global maximum is the maximum over these nine candidates. Integrating over all interim outcomes gives the MSE^∗^. Details can be found in Appendix A.2.

**Figure 2:**
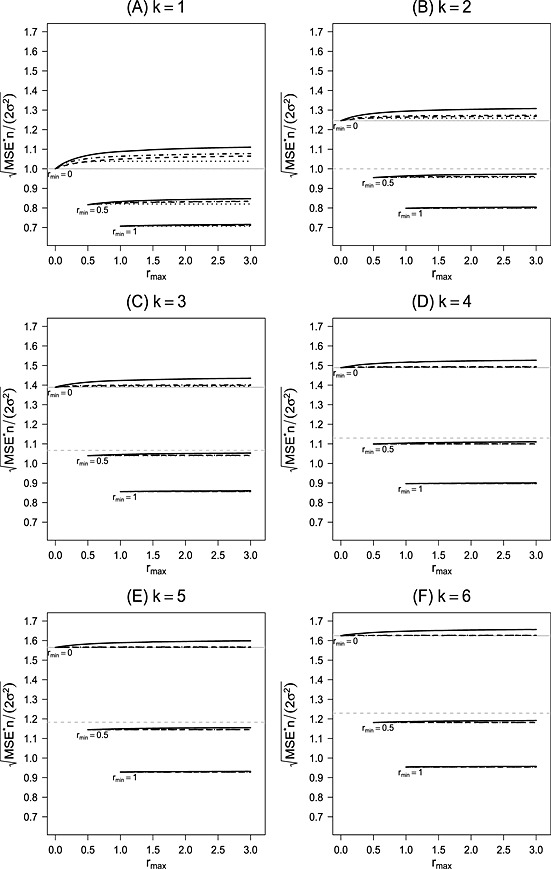
Standardized maximum root mean squared error (RMSE) as a function of *r*_max_ for *r*_min_=0, 0.5, and 1. Values are given for a number of *k* = 1 to 6 treatments in panels (A) to (F). Within one panel, the standardized maximum RMSE is shown for different restrictions on the sample‐size reassessment rule: flexible second‐to‐first‐stage ratios (solid lines), equal second‐to‐first‐stage ratios (dotted lines), restricting $r_{1}\geq r_{0}$ (dot‐dashed lines), and fixing the control (dashed lines). The solid gray horizontal line shows the standardized maximum RMSE for a fixed‐size‐sample test with post‐trial selection. The dashed gray horizontal line shows the standardized RMSE of a fixed‐sample‐size test when selecting the treatment with the maximum effect at the end.

The solid lines in Figure 2 (A) show the maximum RMSE, say RMSE^∗^, divided by the standard error of the first‐stage mean difference, that is, $\sqrt{{\text{MSE}}^{*}}/\sqrt{2\sigma^{2}/n}$. Note that we use here the same standardization as for the maximum Bias and that the standardized RMSE^∗^ does not depend on *n* or *σ*. As for the Bias, for increasing *r*_min_ and decreasing *r*_max_, the standardized RMSE^∗^ is decreasing. Setting *r*_max_=2 and *r*_min_=0, RMSE^∗^ is 1.10 times first‐stage standard error. Increasing *r*_min_ to 0.5 or 1, the values are decreasing to 0.84 and 0.71.

The gray horizontal line through 1 represents the standardized RMSE of the first‐stage mean difference. For the investigated $r_{\min}\geq 0.5,$ the standardized RMSE^∗^ is always smaller than 1, meaning that we gain in precision from the second stage, independently of the sample‐size reassessment rule. If *r*_min_=0, RMSE^∗^ is larger than the first‐stage RMSE, indicating that we can lose in precision if sample sizes are reassessed and the trial can be stopped at interim (compared with a trial that consist of the first stage only). The latter is because of the Bias that is possible under sample‐size reductions and early stopping (Figure 1 (A)).

Setting *r*_min_=*r*_max_>0 gives the RMSE to a fixed‐size sample test with a sample size larger than the first stage. For example, *r*_min_=*r*_max_=0.5, the standardized RMSE is 0.82 decreasing to 0.71 for *r*_min_=*r*_max_=1. It is interesting to see that for $r_{\min}\geq 0.5$, RMSE^∗^ under flexible sample‐size reassessments is only slightly increasing in *r*_max_ and remains close to the RMSE of the fixed‐size‐sample test with second stage per group sample size *r*_min_ *n*. Hence, for sufficiently large *r*_min_, the Bias from any adaptive sample increase will not have a substantial negative effect on the precision of the overall maximum likelihood estimate.

The rows ‘flexible’ in Table 1 shows the standardized RMSE^∗^ when setting *r*_max_=*∞* for *r*_min_=0, 0.5, 1. Without any restrictions on the reassessment rule, setting (*r*_min_,*r*_max_) = (0,*∞*), the standardized RMSE^∗^ is 1.13. Setting *r*_min_=1 and *r*_max_=*∞*, it is 0.72 as compared with 0.71 for the corresponding fixed‐size‐sample test.

#### 3.2.2 Balanced second‐stage sample sizes

Restricting the second‐stage sample size to be balanced between the treatment groups (*r* = *r*_1_=*r*_0_) reduces the CMSE (9) to

(12) $$\text{CMSE}(y,n,\sigma ,r)=\frac{2\sigma^{2}}{n{\left(1+r\right)}^{2}}\left(y^{2}+r\right),$$

where $y=(z_{1}-z_{0})/\sqrt{2}$ is standardand normally distributed. Setting the first derivative to 0, a candidate for the global maximum can be evaluated by *r* ^(1)^=1 − (*z*_1_−*z*_0_)^2^. By calculating the second derivative at the point *r* ^(1)^, it can be shown, that *r* ^(1)^ is a maximum if $|(z_{1}-z_{0})|\leq \sqrt{2}$. This candidate is the global maximum if *r*_min_≤*r* ^(1)^≤*r*_max_; otherwise, the global maximum is achieved for *r* ^(2)^=*r*_min_ or *r* ^(3)^=*r*_max_. The worst case CMSE can be evaluated as the maximum over all three candidates.

(13) $$\tilde{\text{CMSE}}(y,n,\sigma ,r_{\min},r_{\max})=\underset{i=1,2,3:r_{\min}\leq r^{\left(i\right)}\leq r_{\max}}{\max}\text{CMSE}(y,n,\sigma ,r^{\left(i\right)}),$$

and the maximum MSE is obtained by numerical integration

$${\text{MSE}}^{*}(n,\sigma ,r_{\min},r_{\max})=\overset{\infty }{\underset{-\infty }{\int }}\tilde{\text{CMSE}}(y,n,\sigma ,r_{\min},r_{\max})\phi (y)\mathrm{dy}$$

The dotted lines in Figure 2 (A) show the standardized RMSE^∗^ for the case of equal second‐stage sample sizes in the groups. The restriction to balanced sample sizes decreases the RMSE^∗^, the decrease being smaller for larger *r*_min_. Setting *r*_max_=2, the standardized RMSE^∗^ is 1.04, 0.82, and 0.71 for *r*_min_=0, 0.5, or 1, respectively. Note that, for $r_{\min}\geq 0.5,$ the lines corresponding to the different restrictions are indistinguishable. The rows ‘*r*_1_=*r*_0_’ for *k* = 1 in Table 1 show the standardized RMSE^∗^ for *r*_max_=*∞*. Without any restrictions (*r*_min_=0), the standardized RMSE^∗^ is 1.04 and, hence, can still be larger than the RMSE of the first stage. For *r*_min_=0.5 and 1, the standardized RMSE^∗^ is more or less equal to the standardized RMSE of the corresponding fixed‐size‐sample test with per group sample size *r*_min_ *n*. This shows that, for sufficiently large *r*_min_, the worst case Bias from data driven, balanced second‐stage sample size increases has a more or less negligible effect on the precision of the overall maximum likelihood estimate.

#### 3.2.3 Restricting the treatment to $r_{1}\geq r_{0}$

The dot‐dashed lines in Figure 2 (A) show the standardized RMSE^∗^ when restricting the sample size of the treatment group to be as least as large as the sample size of the control group. Setting *r*_max_=2, the standardized RMSE^∗^ is 1.07, 0.83 and 0.71 for *r*_min_=0, 0.5 or 1, respectively, which is only slightly larger than RMSE^∗^ under balanced second‐stage sample sizes. The rows ‘ $r_{1}\geq r_{0}$’ for *k* = 1 in Table 1 shows the maximum for *r*_max_=*∞*. Without any restrictions (*r*_min_=0), the standardized RMSE^∗^ is 1.09. Here, we see some inflation of the RMSE^∗^ compared with the one under the constraint *r*_1_=*r*_0_.

#### 3.2.4 Fixing *r*_0_

Note that, when fixing *r*_0_, the MLE of the control group is unbiased. In the maximization of the CMSE only three of the nine candidates remain. Two candidates are derived by setting *r*_1_=*r*_0_(=*r*_min_) or *r*_1_=*r*_max_. The third candidate can be calculated as candidate *r* ^(6)^ in the maximization of the CMSE with flexible ratios in Appendix A.2. with *r*_min_ replaced by *r*_0_.

The dashed lines in Figure 2 (A) show the standardized RMSE^∗^, assuming *r*_0_=0,0.5, or 1 and *r*_0_≤*r*_1_≤*r*_max_. As expected, the standardized RMSE^∗^ is smaller than the ones with flexible reassessment in the control; however, the difference decreases with increasing *r*_min_. Setting *r*_max_=2, the standardized RMSE^∗^ is 1.06, 0.83, and 0.71 for *r*_0_=0, 0.5, and 1, respectively. We can see from Table 1 and Figure 2 (A) that, for sufficiently large *r*_min_ or large *r*_max_, the differences in RMSE^∗^ between the rule with fixed *r*_0_ and the one with $r_{1}\geq r_{0}$ are only small, so that there is no substantial gain in (minimum) precision from fixing *r*_0_ (the lines in Figure (A) are indistinguishable).

#### 3.2.5 Reshuffling

In case of reshuffling, the CMSE can be rewritten as follows:

(14) $$\text{CMSE}(z_{0},z_{1},v,n_{g},t,\sigma )=\frac{\sigma^{2}}{tn_{g}}\left[{\left(\frac{z_{1}}{1+(1-v)w_{t}}-\frac{z_{0}}{1+vw_{t}}\right)}^{2}+\frac{(1-v)w_{t}}{{\left(1+(1-v)w_{t}\right)}^{2}}+\frac{vw_{t}}{{\left(1+vw_{t}\right)}^{2}}\right],$$

where, as before, $w_{t}=2(\frac{1}{t}-1)$. Recall that the per‐group first‐stage sample size is *t* *n*_*g*_, and the total overall sample size is fixed at 2*n*_*g*_. At the second stage, *v*2(1 − *t*)*n*_*g*_ patients are allocated to the control and (1 − *v*)2(1 − *t*)*n*_*g*_ patients to the treatment group.

By setting the first derivative of (14) to zero, candidates for the global maximum are found. This problem can be reduced to finding the roots of a third‐degree polynomial, and therefore, at maximum three candidates (*v* ^(1)^,*v* ^(2)^,*v* ^(3)^) must be assessed. Note that we did not derive these candidates analytically. Instead, we used the R‐function polyroot 22 for the numerical root finding. Considering furthermore *v* ^(4)^=0 and *v* ^(5)^=1, the worst case CMSE is the maximum over five candidates. Integrating over all interim outcomes gives the maximum MSE, denoted as before by MSE^∗^. Figure (D) in the Appendix gives the subspaces of the interim outcome of treatment and control to evaluate the worst case CMSE. In the white area either *v* ^(1)^, *v* ^(2)^ or *v* ^(3)^ are the global maximizer. As for the maximum Bias, we will, furthermore, also give results when restricting 0≤*v*≤0.5, which means that a larger sample size has to be allocated to the treatment group.

**Figure 3:**
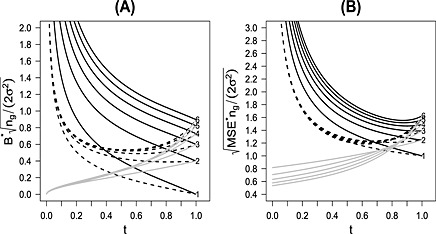
Standardized maximum Bias, panel (A), and the standardized maximum root mean squared error, panel (B), as a function of the timing *t* of the interim analysis for *k* = 1 to 6 treatments compared with one common control for the case of full (solid black lines) and restricted reshuffling (dashed black‐lines). For comparison, the standardized Bias and root mean squared error are given for an adaptive design with treatment selection and a sample size of (1 − *t*)*n*_*g*_(*k* + 1)/2 in the second stage (gray lines).

The solid line marked with 1 (the case *k* = 1) in Figure 3 (B) shows RMSE^∗^ for 0≤*v*≤1 divided by the standard error of a fixed‐size‐sample test with per‐group sample size *n*_*g*_. The standardized RMSE^∗^ is plotted as a function of the timing of the interim analysis *t*. The dashed line marked with 1 gives the corresponding standardized RMSE^∗^ if 0≤*v*≤0.5. Like the Bias, the standardized RMSE^∗^ is decreasing with increasing *t*. For *t* = 0.5, the standardized RMSE^∗^ is 1.39 if 0≤*v*≤1 and decreases to 1.23 if 0≤*v*≤0.5. Note that the standardized RMSE^∗^ is always larger than 1, that is, the RMSE with sample reshuffling (between the experimental and control group) is always larger, and for small *t* substantially larger, than the RMSE of the reference fixed‐sample design with the same overall sample size.

## 4 Multi‐arm trials with interim treatment selection

In this section, we consider two‐stage designs, which start with a control and *k* > 1 experimental treatment groups and where one experimental treatment, say treatment *s*∈{1,…,*k*}, and the control are selected for the second stage. The second stage sample sizes are then set based on the interim results. Again, we assume balanced sample sizes in the first stage, while in some of our rules the second‐stage sample sizes are permitted to be unbalanced.

### 4.1 Maximum Bias

To evaluate the maximum Bias, we search for the selection and sample size adaptation rule that maximize Bias. These are obtained by first maximizing for each MLE, ${\bar{X}}_{i}-{\bar{X}}_{0}$, the conditional Bias (see formula (2) for *k* = 1) with respect to the sample size fractions *r*_*i*_ and *r*_0_ and then selecting the treatment *s* with largest maximized conditional Bias;

(15) $$s=\arg\underset{i=1,\ldots ,k}{\max}\tilde{\text{CB}}\left(z_{0},z_{i},n,\sigma ,r_{\min},r_{\max}\right)$$

Integrating over all interim outcomes gives the worst case Bias;

(16) $${\text{B}}_{k}^{*}\left(n,\sigma ,r_{\min},r_{\max}\right)=\overset{\infty }{\underset{-\infty }{\int }}\ldots \overset{\infty }{\underset{-\infty }{\int }}\tilde{\text{CB}}\left(z_{0},z_{s},n,\sigma ,r_{\min},r_{\max}\right)\phi (z_{k})\ldots \phi (z_{0})dz_{k}\cdots dz_{0},$$

where *s* is data dependently determined as in (15) and *z*_*s*_, *z*_0_ denote the observed interim outcome of the selected treatment and control group, respectively. Note that, for the given *z*_*i*_, each $\tilde{\text{CB}}(z_{0},z_{i},n,\sigma ,r_{\min},r_{\max})$ can be calculated according to the case of *k* = 1 (Section 3.1).

#### 4.1.1 Flexible second‐to‐first‐stage ratios

In case of flexible second‐to‐first‐stage ratios, *r*_0_ is maximized independently from *r*_*s*_ (see formula (2) with *r*_1_ replaced by *r*_*s*_). Because the conditional Bias and thereby also its maximum $\tilde{\text{CB}}$ are increasing in *z*_*s*_ for fixed *z*_0_, we have

(17) $$\underset{i=1,\ldots ,k}{\max}\tilde{\text{CB}}\left(z_{0},z_{i},n,\sigma ,r_{\min},r_{\max}\right)=\tilde{\text{CB}}\left(z_{0},\underset{i=1,\ldots ,k}{\max}z_{i},n,\sigma ,r_{\min},r_{\max}\right).$$

This means that the treatment with the largest worst case conditional Bias at interim is the treatment with the largest observed *z*_*i*_, that is, *z*_*s*_= max*i* = 1,…,*k* *z*_*i*_ and (15) reduces to the selection of treatment

$$s=\arg\underset{i=1,\ldots ,k}{\max}\tilde{\text{CB}}\left(z_{0},z_{i},n,\sigma ,r_{\min},r_{\max}\right)=\arg\underset{i=1,\ldots ,k}{\max}z_{i}$$

The maximum Bias (16) can therefore be reduced to

(18) $${\text{B}}_{k}^{*}\left(n,\sigma ,r_{\min},r_{\max}\right)=\overset{\infty }{\underset{-\infty }{\int }}\overset{\infty }{\underset{-\infty }{\int }}\tilde{\text{CB}}\left(z_{0},z_{s},n,\sigma ,r_{\min},r_{\max}\right)\mathrm{k\Phi}{\left(z_{s}\right)}^{k-1}\phi (z_{s})\phi (z_{0})dz_{s}dz_{0},$$

where *Φ* denotes the cumulative distribution function of the standard normal distribution. Note, the probability density function of the maximum of independent standard normal distributions is *k* *Φ*(*x*)^*k* − 1^ *ϕ*(*x*). Like for *k* = 1, the maximum Bias is independent from *μ*_*i*_ for all *i* = 0,1,…,*k*.

The solid lines in Figure 1 (B) to (F) show the standardized maximum Bias for *k* = 2 to 6 as a function of *r*_max_ for *r*_min_=0, 0.5, and 1. The maximum Bias is standardized by the first‐stage standard error $\sqrt{2\sigma^{2}/n}$ of one treatment‐to‐control comparison. Because of this standardization, the shown Biases are also independent of the first stage sample size *n* and the common variance *σ* ^2^.

The gray horizontal line shows the standardized Bias for a fixed sample size of *n* patients per treatment group and post‐trial selection 20, which results here from setting *r*_min_=*r*_max_=0. Setting *r*_min_=*r*_max_>0 gives an adaptive design where in an interim analysis, one treatment and the control are selected for the second stage and a second stage with a fixed sample size is performed. This means that *r*_min_=*r*_max_ gives the selection Bias without any additional Bias because of sample size reassessment.

As expected, the standardized maximum Bias is increasing with increasing *k*. For flexible reassessment rules, the difference of the maximum Bias to the pure selection Bias (the case *r*_min_=*r*_max_) is large for all shown *k*; however, it is not increasing (rather decreasing) in *k*. Again, the maximum Bias is effectively decreased by increasing *r*_min_.

#### 4.1.2 Maximum Bias under restrictions on the second‐stage sample‐size ratios

Obviously, equality (17) holds also under restrictions like *r*_0_=*r*_*s*_, *r*_0_≤*r*_*s*_ or a fixed *r*_0_. Hence, we can also utilize the mathematical results from Section 3.1 when restricting the second‐to‐first‐stage ratios.

The dotted and the dashed‐dotted lines in Figure 1 (B) to (F) show the standardized maximum Biases for *k* = 2 to 6 with balanced second‐stage sample sizes (*r*_*s*_=*r*_0_) and under the restriction $r_{s}\geq r_{0}$, respectively. Both restrictions substantially reduce the maximum Bias. For k≥2, we see only a small difference between the maximum Bias with balanced sample sizes and the one with the restriction $r_{s}\geq r_{0}$.

The difference of the maximum Bias to the selection Bias (*r*_min_=*r*_max_), that is, the additional Bias due to sample size reassessment is still rather large for *r*_min_=0 but becomes substantially smaller for $r_{\min}\geq 0.5$ and is decreasing with increasing number of treatments *k*. This means that with a larger number of treatments the selection Bias dominates the Bias from data‐driven sample‐size reassessments. Fixing *r*_0_ (dashed lines) leads to a further reduction in the maximum Bias, which is then close to the selection Bias.

#### 4.1.3 Maximum Bias for *r*_max_=*∞*

To investigate the influence of *r*_min_ more carefully, we give in Table 1 the standardized maximum Biases for different *r*_min_, setting *r*_max_=*∞* also for k≥2. For comparison, the rows *r*_min_=*r*_max_ contain the selection Bias without any sample size reassessments. The maximum Bias is decreasing in *r*_min_ also in this fixed sample size case because of the increasing second stage sample sizes. Like for the case *k* = 1, the reduction in the maximum Bias due to an increase in *r*_min_ is more or less independent from the further restrictions on *r*_*s*_ and *r*_0_, and it seems independent from *k*: the maximum Bias always decreases by about 33% when setting *r*_min_=0.5 (compared with *r*_min_=0) and by about 50% for *r*_min_=1.

The table confirms the finding that the restriction $r_{s}\geq r_{0}$ leads to a substantial reduction in the maximum possible Bias, while the restriction to balanced second‐stage sample sizes does not lead to a substantial further reduction. For k≥2 and $r_{\min}\geq 0.5$, fixing *r*_0_ has some (but not a large) additional effect on the maximum Bias and brings the Bias close to the pure selection Bias (*r*_min_=*r*_max_). We may deduce from this findings that a data driven increase in the sample size for the selected experimental treatment group will (when initially large enough) not lead to a substantially additional Bias.

#### 4.1.4 Reshuffling

Like in Section 3.2.5, we assume now that a total sample size of *n*_*g*_ patients per‐group is pre‐planned for the two stages, whereby *t* *n*_*g*_ per‐group are used in the first stage, *t* denoting the timing of the interim analysis. As a consequence, the overall pre‐planned second‐stage sample size is (1 − *t*)*n*_*g*_(*k* + 1). Now, in the interim analysis, one treatment is selected and the second‐stage sample size (1 − *t*)*n*_*g*_(*k* + 1) is reshuffled between the selected treatment and control that means that for some *v*∈(0,1), (1 − *v*)(1 − *t*)*n*_*g*_(*k* + 1) patients are allocated to the selected experimental treatment and *v*(1 − *t*)*n*_*g*_(*k* + 1) patients to the control group. The conditional Bias can be calculated to be (6) with *w*_*t*_=(*k* + 1)/*t* − (*k* + 1). Note that the sample size over both stages is *t* *n*_*g*_+(1 − *v*)(1 − *t*)*n*_*g*_(*k* + 1) in the selected treatment and *t* *n*_*g*_+*v*(1 − *t*)*n*_*g*_(*k* + 1) in the control group. It can be shown that equality (17) holds also in the case of reshuffling (Appendix A.3), so that in the calculation of the maximum Bias ${\text{B}}_{k}^{*}$, the (*k* + 1) dimensional integral can be reduced to a two‐dimensional integral.

Figure 3 (A) shows values of ${\text{B}}_{k}^{*}$ standardized by the standard error of a two‐group fixed‐size‐sample test with sample size *n*_*g*_, that is, $\sqrt{2\sigma^{2}/n_{g}}$. The standardized maximum Bias is shown as function of *t* for *k* = 1 to 6. The solid lines show the values for 0≤*v*≤1 and the dashed lines for 0≤*v*≤0.5. Recall that *v*≤0.5 corresponds to the constraint that the control group is smaller or as large as the selected experimental treatment group. For comparison, the gray solid lines give the maximum Bias for an adaptive design with interim selection of one treatment and control at time point *t*, the second‐stage sample size being (1 − *t*)*n*_*g*_(*k* + 1)/2 per‐group. This is the selection Bias without additional sample size reassessment. The selection Bias is 0 for *t* = 0 because we then perform a fixed‐sample‐size test with only one treatment and control. It is increasing with increasing *t*, being the selection Bias for a trial with post‐trial selection for *t* = 1. This is equivalent to setting *r*_min_=*r*_max_=0 in Figure 1. The standardized maximum Bias (including selection Bias and the Bias due to sample size reassessment) is decreasing with increasing *t* for *v*≤1; however, there is a non‐monotonous behavior for the standardized maximum Bias if restricting *v* to be smaller than 0.5. The maximum Bias is depending on both the selection Bias and the Bias for additional sample‐size reassessment. The selection Bias is increasing with *t*, and the Bias due to sample size reassessment is decreasing with *t*. This leads to a tradeoff between both types of Bias for k≥1.

For *t* = 0.5 and *k* = 2, 3, 4, the standardized maximum Bias is 0.80, 1.00, 1.14 if *v*≤1 and 0.43, 0.50, 0.52 if *v*≤0.5, respectively. For comparison, the selection Bias is 0.23, 0.28, and 0.29 for *k* = 2, 3, and 4. In summary, a sample‐size reshuffling between the selected treatment and the control group can lead to a substantial Bias. The maximum Bias is halved by the constraint that the control group is never larger than the selected experimental treatment group.

### 4.2 Maximum mean squared error

To evaluate the maximum MSE, we proceed similar to evaluating the maximum Bias. The selection rule to maximize the MSE is to select the treatment with the maximum worst case CMSE based on the interim result:

(19) $$s=\arg\underset{i=1,\ldots ,k}{\max}\tilde{\text{CMSE}}\left(z_{0},z_{i},n,\sigma ,r_{\min},r_{\max}\right)$$

Note that the treatment with the maximum worst case CMSE is not necessarily the treatment with the maximum observed *z*_*i*_ at interim or the treatment with the maximum absolute difference to the control |*z*_*i*_−*z*_0_|, because the conditional error (9) cannot be written as a function of *z*_*i*_−*z*_0_. The maximum MSE is a (*k* + 1) dimensional integral over all interim outcomes:

(20) $${\text{MSE}}_{k}^{*}\left(n,\sigma ,r_{\min},r_{\max}\right)=\overset{\infty }{\underset{-\infty }{\int }}\ldots \overset{\infty }{\underset{-\infty }{\int }}\tilde{\text{CMSE}}\left(z_{0},z_{s},n,\sigma ,r_{\min},r_{\max}\right)\phi (z_{k})\ldots \phi (z_{0})dz_{k}\ldots dz_{0},$$

where $\tilde{\text{CMSE}}$ is calculated as discussed in Section 3.2 and *s* is chosen as in (19). To evaluate the (*k* + 1) dimensional integral, numerical integration was performed using the R‐package R2Cuba 21.

#### 4.2.1 Results

Figure 2 (B) to (F) shows the standardized ${\text{RMSE}}_{k}^{*}$ for *k* = 2 to 6. As for *k* = 1, the maximum RMSE was standardized by the standard error of the first stage, that is, $\sqrt{\mathrm{MS}E_{k}^{*}}/\sqrt{2\sigma^{2}/n}$. The solid lines show the scenario with full flexibility on the reassessment rules within the boundary (*r*_min_,*r*_max_), the dashed lines when fixing the sample size of the control group. The dot‐dashed lines show the values when restricting the sample size of the treatment to be larger as the sample size of the control ( $r_{0}\geq r_{s}$) and the dotted lines when restricting the second stage sample size to be balanced (*r*_0_=*r*_*s*_). For comparison, the dashed gray horizontal line shows the standardized RMSE of a fixed‐sample‐size test when selecting the treatment with the maximum effect at the end. The solid gray horizontal line represents the case *r*_min_=*r*_max_=0 where we select after *n* observations per group the treatment with maximum CMSE. By definition (see also formula (9)), for *r*_min_=*r*_max_=0, the CMSE is simply the square of the difference between the estimated and true effect.

Note also that, if we restrict the second stage sample size to be balanced between groups, the treatment with the maximum CMSE at interim is the treatment with the maximum observed ∣*z*_*i*_−*z*_0_∣. Again, the values for *r*_min_=*r*_max_ give the selection RMSE without additional sample size reassessment. We can see from the figures that even though the adaptive sample size reassessment may increase Bias substantially, it has only a small effect on the RMSE, that is, sample‐size reassessments do not increase the RMSE much over the RMSE under treatment selection, at least when sample‐size reductions are limited to $r_{\min}\geq 0.5$. Especially for $r_{\min}\geq 0.5$ lines for the different restrictions are indistinguishable because of the small difference between the results.

Table 1 give the standardized ${\text{RMSE}}_{k}^{*}$ for several scenarios setting *r*_max_=*∞*. Recall that, for a comparison, the rows *r*_min_=*r*_max_ show the RMSE under treatment selection only. Like the maximum Bias, ${\text{RMSE}}_{k}^{*}$ is increasing with increasing *k*; however, for *r*_min_>0, the additional increase in ${\text{RMSE}}_{k}^{*}$ due to sample size reassessment is small in particular under the additional restrictions on *r*_*s*_ and *r*_0_. The difference becomes smaller, the larger *r*_min_ and *k* are. The difference between the fixed and adaptive sample size case is particularly small with balanced second stage sample sizes. This may be due to the fact that balanced sample sizes are optimal with regard to the variance of the second‐stage effect estimate. Moreover, aiming on the reduction of ${\text{RMSE}}_{k}^{*}$, we find for k≥1 that fixing *r*_0_ is not more and can even be less effective than the restriction to balanced sample sizes (in contrast to what we find for the maximum Bias). Again, there is no large difference between the constraints $r_{s}\geq r_{0}$ and *r*_*s*_=*r*_0_, in particular, for larger *k*.

#### 4.2.2 Reshuffling

For the case of a sample‐size shuffling between the selected treatment and the control group, the maximum conditional mean squared error, $\tilde{\text{CMSE}}$, can be calculated as in Section 3.2.5. Figure 3 (B) shows the resulting standardized ${\text{RMSE}}_{k}^{*}$ for *k* = 1 to 6 for 0≤*v*≤1 (solid black lines) and for the restriction 0≤*v*≤0.5 (dashed black lines). The gray solid lines gives the maximum selection RMSE for an adaptive design, selecting one treatment and control at interim time point *t*, allocating in the second stage (1 − *t*)*n*_*g*_(*k* + 1)/2 patients to each of the two groups. In this balanced case, the treatment with the maximum CMSE at interim is the treatment with the maximum absolute observed difference |*z*_*i*_−*z*_0_| at interim. Note again that this is not necessarily true if we allow for reshuffling leading to unbalanced second stages.

The selection RMSE (gray lines) is increasing with increasing *t*. This is similar to the results of 20 where the selection Bias was calculated for the case of selecting the treatment with maximum effect at interim. We note again that selecting the treatment with the maximum treatment effect is not the same as selecting the treatment with the maximum CMSE at interim. As for the Bias, the standardized ${\text{RMSE}}_{k}^{*}$ shows a non‐monotonous behavior. There is a trade‐off between the variance, which is increasing with *t* for selection and the Bias due to sample size reassessment, which is decreasing with *t*. For k≥2 and with the constraint *v*≤0.5, there is a *t* for which the RMSE is minimal. The minimum is achieved at *t*‐values close to 0.5.

The later the interim analysis the smaller the difference between ${\text{RMSE}}_{k}^{*}$ and selection RMSE. For *t* = 0.5 and *k* = 2, 3, 4, the standardized maximum Bias is 1.56, 1.67, 1.74 if *v*≤1 and 1.28, 1.27, 1.25 if *v*≤0.5. For a comparison, the selection RMSE is 0.99, 0.93, 0.88 for *k* = 2, 3, and 4, respectively. Note that the case *t* = 1 gives the worst case RMSE of a classical fixed‐sample‐size parallel group design where the single treatment is selected post‐trial (in a fully flexible manner), and that the ${\text{RMSE}}_{k}^{*}$ of an adaptive design with mid‐trial treatment selection and sample size reshuffling is smaller.

## 5 Discussion

We investigated in this paper the maximum effect of data‐driven sample‐size reassessments and treatment selection on Bias and precision of maximum likelihood estimators in multi‐armed adaptive designs. We assumed that in an interim analysis, one out of *k* treatments and the control are selected for a second stage and sample sizes are reassessed in a fully flexible manner with and without restrictions. To best of our knowledge, we are the first who consider Bias and MSE under flexible selection and sample size reassessment rules. In 20, for instance, selection Bias and MSE were considered without sample size reassessment and only for some specific selection rules.

To cope with flexible decision rules, we calculated the maximum Bias and maximum MSE searching at each possible interim outcome for the worst case treatment selection and sample size assignments, which maximize the conditional Bias or conditional MSE. We are aware of the fact that the determination of maximum Bias and MSE will lead to an overestimation and that Bias and MSE may in reality be (substantially) smaller. To bound the conservatism of our approach, we considered several restrictions on the sample‐size rules, like balanced second‐stage sample sizes or to rules for which the selected experimental treatment group is as least as large as the control group. We saw that these restrictions substantially reduce the maximum Bias and maximum MSE and that in some cases (e.g. when *k* = 1 and *r*_min_=1) the maximum Bias and maximum inflation of the MSE is small enough to justify the use of the MLE.

In spite of the conservatism of our approach, we have been able to draw several important conclusions. One important conclusion is that a lower bound for the second stage sample sizes may effectively reduce Bias and inflations of the MSE. We saw, for instance, that under the constraint that the second stage sample sizes are at least as large as the first stage *n* (i.e. the case *r*_min_=1); Bias is in general limited and not much larger than the pure selection Bias. This is particularly the case under the restriction that in the second stage sample the treatment group is as least as large as the control group. Moreover, we found that the maximum Bias is not much further decreased by forcing the treatment groups to be balanced at the second stage or the size of the control group to be fixed. Constraining the second stage sample sizes to be at least as large as the first stage *n* has an even more pronounced effect on the maximum MSE, which is more or less independent from the maximum sample size (*r*_max_) and the additional restriction on the second‐stage allocation ratios. We can, therefore, conclude that with a sufficiently large minimal second‐stage sample size a further increase of the sample size in the selected treatment group has only a limited negative effect on Bias and MSE.

We also learned that when fixing the total sample size and reshuffling the (fixed) second‐stage sample size between the control and selected treatment group the additional Bias and MSE due to the sample‐size reassessments may be substantial even under the (realistic) constraint that the control group is not larger than the experimental treatment group. This is particularly the case when the interim analysis is done early. Note that the results with fixed and flexible overall sample sizes are not easy to compare because we had to use different standardizations for the reshuffling and the other cases, and because in the other cases, the total sample size is not fixed but data dependent and of an less determined magnitude.

Our paper necessarily leaves important questions open. It is known that the selection Bias can be severe even without sample‐size reassessments if selection is done late. Early selection in general will reduce the Bias as compared with ‘post‐trial’ selection 20. Our findings confirm these results. Hence, an important question, that goes beyond the scope of this paper, is the performance of adjusted estimates to account for the selection Bias under flexible selection and sample‐size reassessments. To this end, it is important to note that Bias adjusted estimates have only been suggested and considered for designs with fixed (known) selection rules, namely selecting the seemingly most efficient treatment. We consider shrinkage estimates as one of the most interesting candidates as they are known to perform well in terms of the MSE under the common treatment selection process (compared with 19) but other estimates may be considered as well. Another interesting and important extension of our work would be the consideration of selections rules with more than one selected experimental treatment and with realistic constraints on the selection process. Selection of more than one treatment for the play‐the‐winner rule without additional sample‐size reassessment was investigated in 20. Calculation of the maximum Bias or MSE for further selection rules would be an interesting contribution.

## Figures and Tables

**Figure A1:**
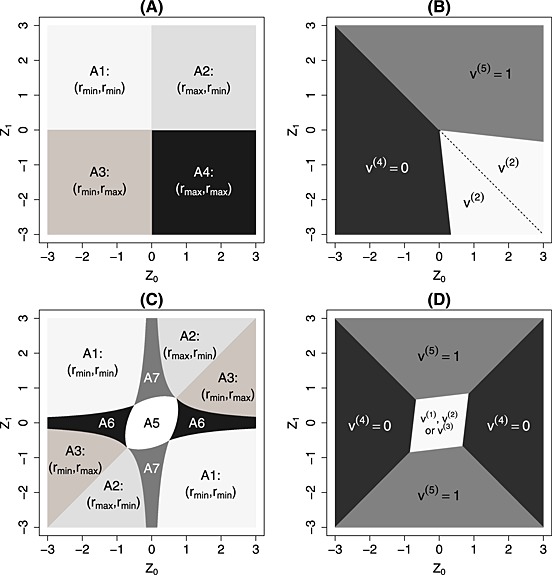
Subsets of the interim outcome of treatment and control (*z*_0_,*z*_1_) to be used for evaluating the worst case conditional Bias (first row) and the worst case conditional MSE (second row). Subsets are given for flexible second‐to‐first‐stage ratios (first column) and the case of reshuffling (second column).

## Appendix 1

### 1.1

#### A.1 Calculation of the CMSE

For the calculation of the conditional mean squared error, we set $Z_{\left(i,j\right)}=({\bar{X}}_{\left(i,j\right)}-\mu_{i})\sqrt{n/\sigma^{2}}$ for *i* = 0,1 and *j* = 1,2.

$$\begin{array}{c} \text{CMSE}(z_{0},z_{1},r_{0},r_{1},n)=\\ =\text{E}\left[{\left(\left({\bar{X}}_{1}-{\bar{X}}_{0})-(\mu_{1}-\mu_{0}\right)\right)}^{2}\left|\;Z_{\left(i,1\right)}=z_{i},i=0,1\right.\right]\\ =\text{E}\left[{\left(\frac{\frac{\sigma }{\sqrt{n}}Z_{\left(1,1\right)}+\frac{r_{1}\sigma }{\sqrt{n_{\left(1,2\right)}}}Z_{\left(1,2\right)}}{1+r_{1}}-\frac{\frac{\sigma }{\sqrt{n}}Z_{\left(0,1\right)}+\frac{r_{0}\sigma }{\sqrt{n_{\left(0,2\right)}}}Z_{\left(0,2\right)}}{1+r_{0}}\right)}^{2}\left|\;Z_{\left(i,1\right)}=z_{i},i=0,1\right.\right]\\ =\text{E}\left[{\left(\frac{\frac{\sigma }{\sqrt{n}}Z_{\left(1,1\right)}}{1+r_{1}}-\frac{\frac{\sigma }{\sqrt{n}}Z_{\left(0,1\right)}}{1+r_{0}}\right)}^{2}+2\left(\frac{\frac{\sigma }{\sqrt{n}}Z_{\left(1,1\right)}}{1+r_{1}}-\frac{\frac{\sigma }{\sqrt{n}}Z_{\left(0,1\right)}}{1+r_{0}}\right)\left(\frac{\frac{r_{1}\sigma }{\sqrt{nr_{1}}}Z_{\left(1,2\right)}}{1+r_{1}}-\frac{\frac{r_{0}\sigma }{\sqrt{nr_{0}}}Z_{\left(0,2\right)}}{1+r_{0}}\right)\right.\\ \left.\;\;+{\left(\frac{\frac{r_{1}\sigma }{\sqrt{nr_{1}}}Z_{\left(1,2\right)}}{1+r_{1}}-\frac{\frac{r_{0}\sigma }{\sqrt{nr_{0}}}Z_{\left(0,2\right)}}{1+r_{0}}\right)}^{2}\left|\;Z_{\left(i,1\right)}=z_{i},i=0,1\right.\right]\\ =\frac{\sigma^{2}}{n}\left[{\left(\frac{z_{1}}{1+r_{1}}-\frac{z_{0}}{1+r_{0}}\right)}^{2}+\frac{r_{1}}{{\left(1+r_{1}\right)}^{2}}+\frac{r_{0}}{{\left(1+r_{0}\right)}^{2}}\right] \end{array}$$

because

$$\text{E}\left[{\left(\frac{\frac{r_{1}\sigma }{\sqrt{nr_{1}}}Z_{\left(1,2\right)}}{1+r_{1}}-\frac{\frac{r_{0}\sigma }{\sqrt{nr_{0}}}Z_{\left(0,2\right)}}{1+r_{0}}\right)}^{2}\left|\;Z_{\left(i,1\right)}=z_{i},i=0,1\right.\right]=\frac{\sigma^{2}}{n}\left(\frac{r_{1}}{{\left(1+r_{1}\right)}^{2}}+\frac{r_{0}}{{\left(1+r_{0}\right)}^{2}}\right)$$

and

$$\text{E}\left[\frac{\frac{r_{1}\sigma }{\sqrt{nr_{1}}}Z_{\left(1,2\right)}}{1+r_{1}}-\frac{\frac{r_{0}\sigma }{\sqrt{nr_{0}}}Z_{\left(0,2\right)}}{1+r_{0}}\left|\;Z_{\left(i,1\right)}=z_{i},i=0,1\right.\right]=0$$

#### A.2 Maximizing the CMSE

To maximize the CMSE (9) for given *z*_0_ and *z*_1_ at interim, nine candidates have to be investigated. Apparently, candidates $\left(r_{0}^{\left(1\right)},r_{1}^{\left(1\right)})=(r_{\min},r_{\min}\right)$, $\left(r_{0}^{\left(2\right)},r_{1}^{\left(2\right)})=(r_{\max},r_{\min}\right)$, $\left(r_{0}^{\left(3\right)},r_{1}^{\left(3\right)})=(r_{\min},r_{\max}\right)$ and $\left(r_{0}^{\left(4\right)},r_{1}^{\left(4\right)})=(r_{\max},r_{\max}\right)$ have to be investigated. By setting the first derivative (with respect to *r*_0_ and *r*_1_) to 0 and solving the corresponding system of two equations, candidate (5) can be assessed with

$$\left(r_{0}^{\left(5\right)},r_{1}^{\left(5\right)}\right)=\left(\frac{-1+2z_{0}^{2}-z_{0}z_{1}+z_{1}^{2}}{-1+z_{0}z_{1}+z_{1}^{2}},\frac{-1+2z_{1}^{2}-z_{0}z_{1}+z_{0}^{2}}{-1+z_{0}z_{1}+z_{0}^{2}}\right).$$

Depending on the given *z*_1_ and *z*_0_, this candidate is either a minimum or a maximum. If a maximum, this candidate is ineligible if either $r_{0}^{\left(5\right)}$ or $r_{1}^{\left(5\right)}$ is larger than *r*_max_ or smaller than *r*_min_. Setting $r_{0}^{\left(6\right)}=r_{\min}$, the corresponding worst case reassessment rule for the treatment group can be calculated by setting the first derivative with respect to *r*_1_ (assuming *r*_0_ fixed) to zero and results in

$$\left(r_{0}^{\left(6\right)},r_{1}^{\left(6\right)}\right)=\left(r_{\min},\frac{1+r_{\min}+2z_{0}z_{1}-2z_{1}^{2}-2r_{\min}z_{1}^{2}}{1+r_{\min}-2z_{0}z_{1}}\right)$$

Candidates $\left(r_{0}^{\left(7\right)},r_{\min}\right)$, $\left(r_{\max},r_{1}^{\left(8\right)}\right)$ and $\left(r_{0}^{\left(9\right)},r_{\max}\right)$ can be assessed similarly. The global maximum for given *z*_0_ and *z*_1_ is the maximum over all nine candidates and formula (10) can be rewritten as.

(21) $$\tilde{\text{CMSE}}\left(z_{0},z_{1},n,\sigma ,r_{\min},r_{\max}\right)=\underset{i=1,...,9:r_{\min}\leq r_{0}^{\left(i\right)},r_{1}^{\left(i\right)}\leq r_{\max}}{\max}\left(\text{CMSE}\left(z_{0},z_{1},n,\sigma ,r_{0}^{\left(i\right)},r_{1}^{\left(i\right)}\right)\right)$$

Evaluating $\tilde{\text{CMSE}}$ for all possible (*z*_0_,*z*_1_) and integrating over all interim outcomes gives MSE^∗^.

Figure (C) in the Appendix shows the subsets (corresponding to the several candidates) when setting *r*_min_=0 and *r*_max_=*∞*. The subset A5 is the area where candidate 5 is the global maximum. See also the subsets for candidates 1 (area A1), 2 (area A2), 3 (area A3), 6 (area A6), and 7 (area A7). It can be seen that candidates 4, 8, and 9 are no global maxima. Some of the regions are similar to the regions maximizing the conditional Bias (Figure A). For *z*_1_ or *z*_0_ close to 0, the worst‐case reassessment rule to maximize the CMSE is different from setting *r*_0_ or *r*_1_ to *r*_min_ or *r*_max_.

#### A.3 Maximum CB under reshuffling

The following equality holds in the case of reshuffling:

(22) $$\underset{i=1,\ldots ,k}{\max}\tilde{\text{CB}}\left(z_{0},z_{i},n_{g},t,\sigma \right)=\tilde{\text{CB}}\left(z_{0},\underset{i=1,\ldots ,k}{\max}z_{i},n_{g},t,\sigma \right).$$

For fix *v*, *n*_*g*_, *t*, and *z*_0_, the conditional Bias (6) is monotonous in *z*_*i*_. Assume now that, for some observed *z*_*s*_ and *z*_0_, the optimal second‐stage allocation rate is $\tilde{v}$. For a $z_{s}^{*}>z_{s}$, because of monotonicity, $\text{CB}(z_{0},z_{s}^{*},\tilde{v},n_{g},t,\sigma )>\text{CB}(z_{0},z_{s},\tilde{v},n_{g},t,\sigma )$. $\tilde{v}$ may not be the allocation rate maximizing the conditional Bias for $z_{s}^{*}$, but finding the actual optimum ${\tilde{v}}^{*}$ can only increase the Bias and therefore $\text{CB}(z_{0},z_{s}^{*},{\tilde{v}}^{*},n_{g},t,\sigma )\geq \text{CB}(z_{0},z_{s}^{*},\tilde{v},n_{g},t,\sigma )$ concluding that $\tilde{\text{CB}}$ is monotonous in *z*_*i*_ (for fixed *z*_0_). Therefore, equality (22) holds.
